# A Realistic, Low-Cost Simulated Automated Chest Compression Device

**DOI:** 10.21980/J8M63C

**Published:** 2024-04-30

**Authors:** Jessica Joyce, Elyse Fults, Julia Rajan, Alexandra Plezia, Carolyn Clayton, Sara M Hock

**Affiliations:** * Rush University Medical Center, Rush Medical College, Chicago IL; ^ Rush University Medical Center, Department of Emergency Medicine, Chicago, IL; † Midwestern University, Chicago College of Osteopathic Medicine, Downers Grove, IL; ** Loyola University Chicago, Stritch School of Medicine, Maywood, IL

## Abstract

**Audience:**

This simulated automated chest compression device was designed for use in simulation cardiac arrest cases involving emergency medicine residents, but it would be applicable to other learners such as nurses, pharmacists, and medical students.

**Background:**

Automated chest compression devices (ACCD) are commonly utilized in cardiac arrest in the emergency department and by emergency medical services (EMS) as patients arrive in the ED.^1^ Prolonged simulated cardiac arrest can be challenging to maintain proper chest compression depth and technique.^2^ Resident learning may be enhanced during cardiac arrest in the simulation environment by implementing the use of a simulated ACCD.

**Educational Objectives:**

By the end of this educational session using a resuscitation trainer or high-fidelity manikin, learners should be able to:

1. Recognize appropriate application of simulated ACCD to an ongoing resuscitation case
2. Demonstrate proper positioning of simulated ACCD in manikin model
3. Integrate simulated ACCD to provide compressions appropriately throughout cardiac arrest scenario

**Educational Methods:**

We developed a cost-effective simulated ACCD for use in resuscitation simulation cases. An initial pilot session identified components of fidelity that were used to model the simulated ACCD after those utilized in clinical situations. Three simulated devices were created and then tested for efficacy during high-fidelity simulation with 25 emergency medicine residents.

**Research Methods:**

Visual analog scales were used to explore how the simulated ACCD affected perceived realism and stress level during the cardiac arrest simulation. Qualitative data were collected through open-ended learner feedback comments. The institutional review board at our institution reviewed this project and determined that it was exempt.

**Results:**

With inclusion of the simulated ACCD device, learners rated the simulation “more realistic” with an average rating of 74/100 and “less stressful” with an average rating of 69/100 on the visual analog scales. Learner comments noted that the use of the ACCD in simulation resulted in better resource availability and accurate environmental noise.

**Discussion:**

The simulated ACCD presented here was found to be effective, realistic, and practical for use by learners in a resuscitation curriculum. Our results suggest that implementating a cost-effective simulated ACCD ($98 for supplies) in high-fidelity simulation cardiac arrest cases enhances the perceived realism of the environment and offers physician learners a low-stress opportunity to practice the clinical application of ACCD in cardiac arrest resuscitation. Additionally, the use of the simulated ACCD, specifically in a prolonged resuscitation, eliminated the need for physically demanding manual chest compressions. Anecdotally, in simulated environments we have observed poor-quality manual chest compressions due to an understanding that the manikin is “not real,” leading to decreased psychological fidelity from the shared acceptance of the poor-quality compressions. Thus, the presence of a simulated clinical device providing chest compressions could have increased the feel of realism through improved psychological fidelity. Additionally, we note that the physical and psychological fidelity of this simulated device was sufficient for physicians to perceive clinical implementation, but may be suboptimal for assistive staff, who are focused on the specific functionality and may benefit from training on the physical device in clinical use. Finally, our simulated ACCD resembles the clinical device our department uses; we advise modifications as appropriate to allow a simulated ACCD created for other learners to also resemble their clinically used ACCD.

**Topics:**

Automated chest compression device, ACLS, improvised equipment, high fidelity simulation.

## USER GUIDE

[Table t1-jetem-9-2-i7]

**Table t1-jetem-9-2-i7:** 

List of Resources:	
Abstract	7
User Guide	9
Learner Materials	14

**Learner Audience:**

Interns, junior residents, senior residents, emergency nurses, pharmacists, medical students, paramedic students, allied health professionals.

**Time Required for Implementation:**

Construction of the simulated ACCD takes approximately 6–8 hours for one device. Learners familiar with clinical ACCD use would require about 1–2 minutes for introduction to the simulated functionality of the device prior to implementation in simulated scenarios; learners unfamiliar with the ACCD would require about 10–20 minutes.

**Recommended Number of Learners per Instructor:**

We recommend four to six physician learners for high-fidelity cardiac arrest cases. When including multidisciplinary learners (eg, RN and PharmD), one could reasonably increase learners to eight per case for realism of cardiac arrest scenarios.

**Topics:**

Automated chest compression device, ACLS, improvised equipment, high fidelity simulation.

**Objectives:**

By the end of this educational session using a resuscitation trainer or high-fidelity manikin, learners should be able to:

1. Recognize appropriate application of simulated ACCD to an ongoing resuscitation case
2. Demonstrate proper positioning of simulated ACCD in manikin model
3. Integrate simulated ACCD to provide compressions appropriately throughout cardiac arrest scenario

### Linked objectives and methods

Emergency medicine residents often practice crisis resource management and specifically cardiac arrest resuscitation leadership in simulated settings prior to engaging in these roles clinically. Increased availability of automated chest compression devices (ACCDs) makes practicing the use of such devices a critical component of simulation in resuscitation cases. Studies have demonstrated that mechanical chest compressors may increase the rate of return of spontaneous circulation (ROSC) and 30-day survival, further demonstrating the likelihood of increasing ACCD use in future clinical practice.^1^ Trainees will recognize the utility of the ACCD and request its implementation by the resuscitation team (Objective 1).

Clinical efficacy of the ACCD depends on proper use and positioning. The physical fidelity of the model allows the learner to achieve proper placement of the device on the manikin torso. (Objective 2).

In addition, learners find it difficult to maintain the effort required for high-quality CPR during prolonged simulated resuscitations, often leading to poor effort or limited compression depth. Indeed, learners in simulation are inaccurate in their self-assessment of compression depth.^4^ In a clinical scenario, this would necessitate cardiac arrest leader intervention through a change of compressors. However, in the simulated setting such “realism fatigue” creates a mental logic loop of “poor compressions - change compressor-discard reasoning due to simulation.” This adds to cognitive load, defined as the limited capacity and duration of working memory required for learning novel information.^5^ Learners in a simulated ACCD scenario could then utilize the simulated ACCD throughout the resuscitation, including with pauses when appropriate. (Objective 3). Being able to rely on the simulated ACCD will improve the cognitive load for the leader of the cardiac arrest.

Finally, in simulated scenarios where ancillary staff are not readily available to participate, physician learners who do not usually perform chest compressions in a clinical setting will be relieved of this duty to continue reasoning through the cognitive leadership of the resuscitation.

### Recommended pre-reading for instructor

Instructors should be familiar with the use of an ACCD in a cardiac arrest. Instructional guides for various ACCD models may be found online (eg, LUCAS^®^ 3 Chest Compression System, ZOLL AutoPulse Resuscitation System Model 100).^6^,^7^

### Learner responsible content (LRC)

We suggest the same content as for instructors, as above. Learners should be familiar with the American Heart Association adult cardiac arrest algorithm.^8^

### Implementation Methods

A low-cost simulated ACCD may increase comfort and implementation of clinical ACCDs, which are shown to improve outcomes of cardiac arrest.^1^, ^3^ In this innovation, we constructed a simulated ACCD for use in simulated adult resuscitation cases with physical, conceptual, and psychological fidelity components.

The simulated ACCD requires an adult manikin torso to allow the conceptual fidelity of simulated compression in the correct anatomic location. This simulated trainer requires little orientation to learners familiar with a clinical ACCD due to its physical and functional similarity. For our learners’ purposes, we used a high-fidelity manikin with physiologic vital signs and pulses to allow the completion of several cardiac arrest resuscitation scenarios. Psychological safety in simulation was achieved using our standard pre-briefing information which includes a baseline statement appreciating the learner’s knowledge base, encouraging treatment of the simulation as an authentic clinical situation, and ensuring that occurrences during the simulation remain confidential.

The didactic portion of the training required for the simulated ACCD should include a pre-briefing orientation to the functional steps of the simulated device. These steps include positioning of the board, clipping the attached top, powering on the device, positioning the top piece of the device above the patient and using the start/pause buttons. The estimated time for staff familiar with a clinical ACCD is 1–2 minutes. If staff are unfamiliar with a commercially available ACCD, we expect this training to take 10–20 minutes and recommend the presence of the clinical ACCD for comparison. The remainder of the debriefing time spent following the simulation cases could focus on the overall clinical case with a focus on the integration of the simulated ACCD into the flow of the case.

### List of items required to replicate this innovation

1. CPR full body low-fidelity manikin or full body high-fidelity manikin (eg, Gaumard Hal S3201). Estimated cost $1800–$50,000.
2. One 20 gallon trash can (eg, Rubbermaid BRUTE 20 gal. round vented trash can). Estimated cost $30.
3. Blank skateboard deck 32” × 8” (eg, rabd blank skateboard deck purchased from Amazon). Estimated cost $30.
4. An accordion toilet plunger (eg, Master Plunger MPS4). Estimated cost $10.
5. Portable wireless speaker (eg, HOAIYO speaker purchased from Amazon). Estimated cost $10.
6. Silicone vacuum suction cup (eg, therapy massage set purchased from Amazon). Estimated cost $8.
7. Plastic pitcher (eg, 32oz Medline Gray Plastic Pitcher). Estimated cost $1.
8. Double roller cupboard cabinet door latch (available at Home Depot). Estimated cost $2.50.
9. Thick rubber elastic band (eg, Goody hair band). Estimated cost $0.20.
10. Four shades of acrylic paint (eg, Americana acrylic paint 2oz). Estimated cost $6.
11. Bubble cushioning wrap. Estimated cost $0.50.
12. Glue gun and sticks (eg, ArtMinds high temperature glue gun). Estimated cost $10.
13. Drill (eg, RYOBI One+ Cordless drill) Estimated cost $60.
14. Hand saw (eg, Stanley 4.5in Tooth Saw). Estimated cost $17.
15. Wood saw (eg, Stanley 15in). Estimated cost $20.
16. Block sanding sponge (eg, 3M medium grip). Estimated cost $6.
17. Automated Chest Compression Device (eg, LUCAS, AutoPulse) to obtain realistic audio to play via speakers. Estimated cost $16,000.

### Approximate cost of items to create this innovation

The materials utilized to create the simulated ACCD total approximately $100 USD, with additional cost for tools if needed of $107. Time required for creation of one simulated ACCD is approximately 8 hours. If available, the use of a full body manikin simulator is ideal. If not already purchased, these are available from multiple sources and range in cost from $1800–50,000 USD. Manikins can be used in many additional settings after purchase.

### Detailed methods to construct this innovation

**Figure 1:**
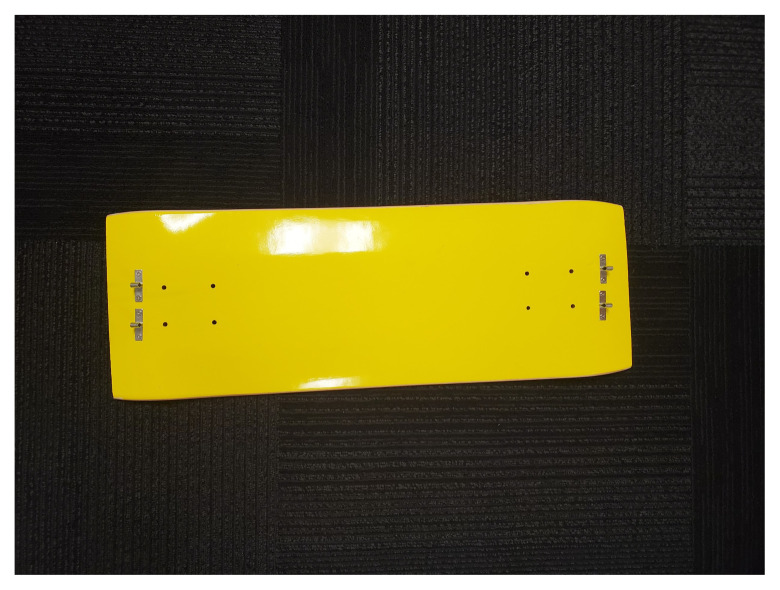
Two latch clips are arranged on each side of the skateboard. They are attached to the skateboard with screws. This simulates the back plate of an ACCD.

1. The skateboard is utilized as the backboard piece of the simulated ACCD. The rounded ends of the skateboard, approximately four inches in length, are removed using a wood saw. The ends are smoothed using the sanding sponge. Four latch clips are screwed into the board in parallel as demonstrated in Figure 1. We recommend measuring the manikin(s) planned for use with the simulated ACCD to ensure the appropriate width of clips. Retaining extra width of the skateboard (approximately 24”) can allow for adjustments as needed during construction. Our latch width was 20” for a manikin torso measuring 18”×10.”
2. The top piece of the trash can is utilized as the top part of the simulated ACCD. This piece, approximately 8” wide and 200 degrees of the circumference, including the handle pieces, is cut off using the hand saw. Two latches are attached to the inner sides of the piece for attachment to the backboard.[Fig f2-jetem-9-2-i7][Fig f3-jetem-9-2-i7]
3. The bottom two-thirds of the plunger is removed with scissors. The top rounded part of the silicone vacuum suction cup is then pushed into the bottom section of the plunger, for a snug fit.[Fig f4-jetem-9-2-i7]
4. Using a large drill bit, a 2-inch diameter hole is made in the top piece for insertion of the plunger. The plunger is secured using a thick rubber elastic hair tie placed around the plunger handle. The tie is then wrapped around 2 screws placed approximately 1 inch away from each side of the hole. A latch clip is glued 1 inch in front of the hole. The bulbs on the handle of the plunger allow for adjustment of the height with the tension of the elastic band maintaining that height.[Fig f5-jetem-9-2-i7]
5. The handle of the pitcher is removed with the hand saw. A latch is glued to the inner side of the pitcher for attachment to the top piece over the plunger handle. This feature allows for modification of the plunger/tie fixation if needed.[Fig f6-jetem-9-2-i7]
6. Bubble cushioning wrap is cut to fit the top piece of the pitcher and glued with the glue gun. Three buttons are painted on cardstock to represent power on/off, a play feature, and a pause feature. The cardstock is secured to the top of the pitcher.[Fig f7-jetem-9-2-i7]
7. Audio of a clinical automated chest compression device is obtained using a phone recording application and saved for use with the wireless speaker. A mobile device is connected to the wireless speaker to play audio. The speaker is placed near the manikin for use in the simulation case.[Fig f8-jetem-9-2-i7]

### Results and tips for successful implementation

A total of three devices were made to allow multiple groups to participate in education simultaneously. Our initial pilot with a subset of two learners helped determine the critical components for physical and psychological fidelity, which were the device audio cues, the side clipping mechanism, sequential button engagement, and the presence of a suction device in adjusting to the size of the patient. This informed modifications to the simulated ACCD device presented here.

The simulated ACCD device was tested in July 2023 during high-fidelity simulation as a component of cardiac resuscitation education for 25 emergency medicine residents. Additional learners were present during our instruction, including several medical students and nurses; data collection focused on the resident learners. During this simulation curriculum, the three simulated ACCD were successfully integrated into repeated cardiac arrest simulation cases and survey data regarding realism was collected from resident learners.

A total of 25 learners surveyed reported their level of training as PGY1 (48%), PGY2 (32%), PGY3 (20%), 48% Male, and reported prior participation in less than 5 codes (44%), 5–10 codes (20%), or more than 10 codes (36%).

Realism and stress level of cases involving the simulated ACCD were evaluated using a visual analog scale ranging from much less real (0) to much more real (100) and much more stressful (0) to much less stressful (100). The visual analog scale scores were measured in mm and converted to scores 1–100. Learners favored “more realistic” as a descriptor for the simulated ACCD as demonstrated by the average rating of 74 on the scale. Overall, learners reported less stress with an average rating of 69. Of note, the generalizability of this feedback could be limited due to learner experience level.

Learner feedback included comments that the simulated ACCD improved resource availability and contributed accurate environmental noise.

### Associated Content

- **Learner Materials**
  ○ Pre-Curriculum Survey
  ○ Post Survey

## Figures and Tables

**Figure f9-jetem-9-2-i7:**
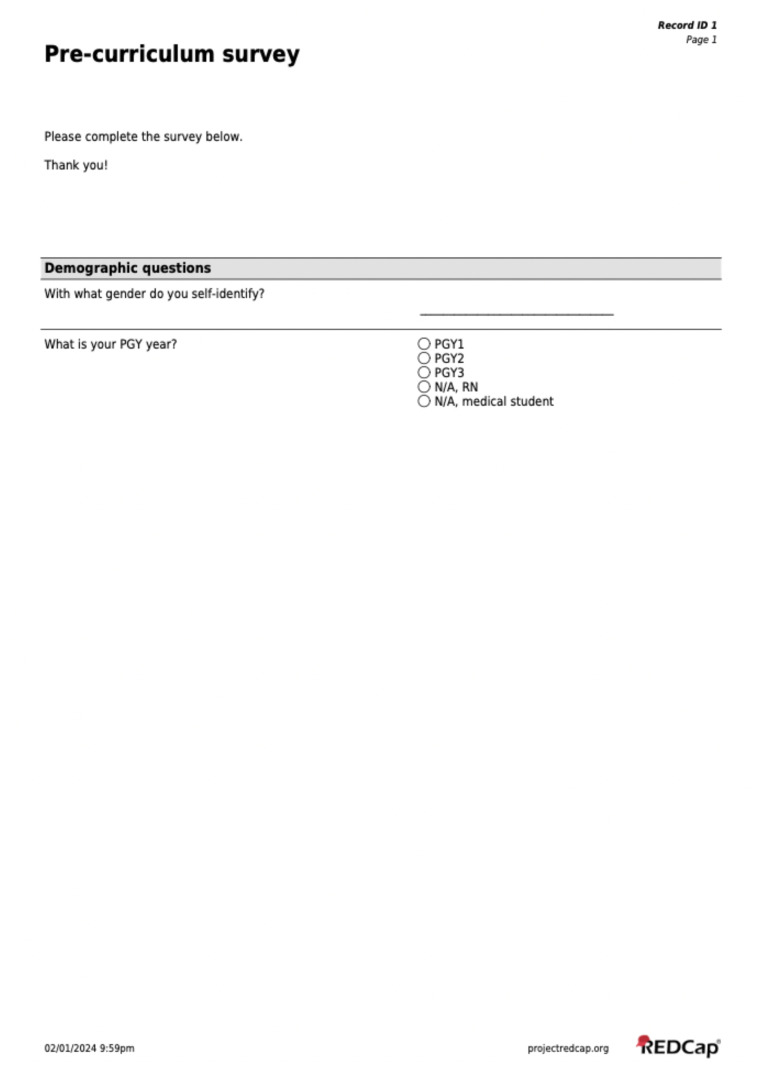


**Figure f10-jetem-9-2-i7:**
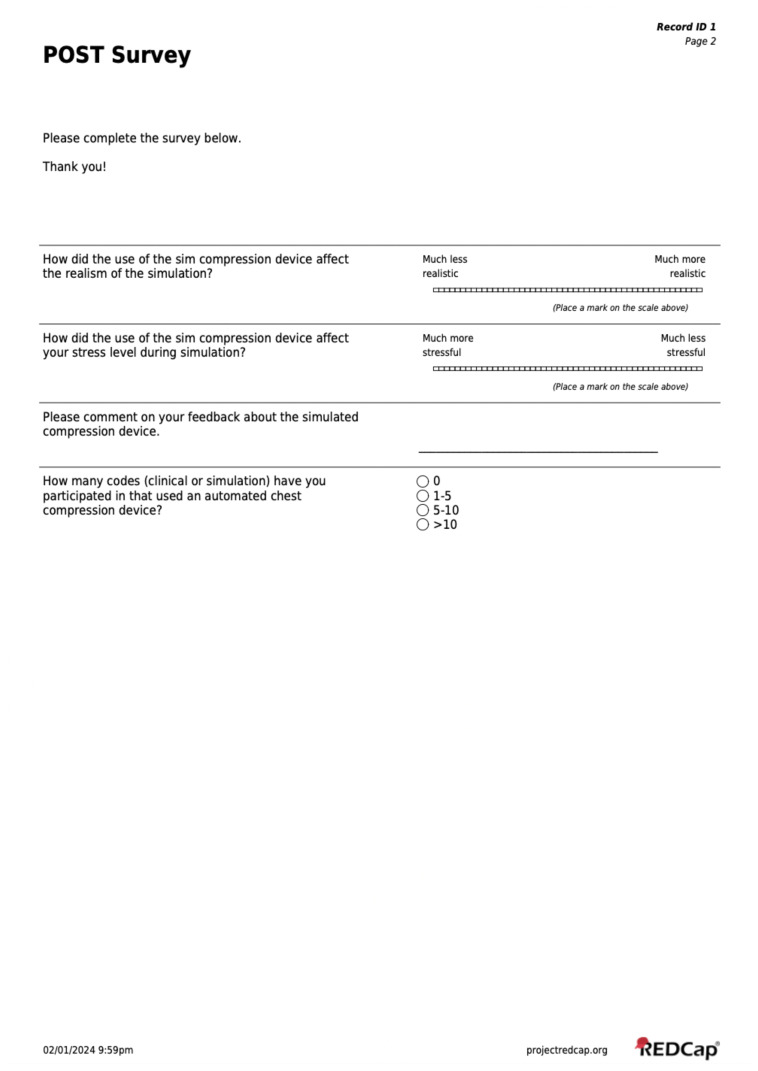


**Figure 2 f2-jetem-9-2-i7:**
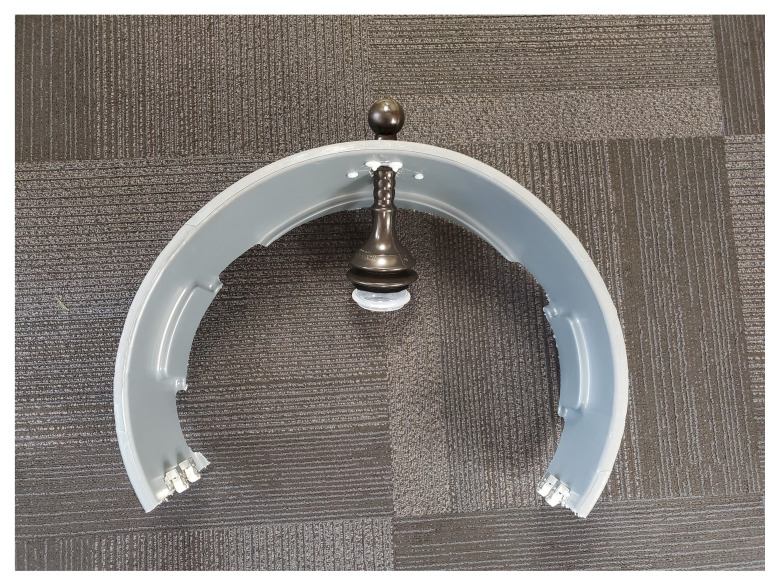
The top rim of a trash can is cut using a hand saw. An arch is formed by removing 160 degrees of the rim’s circumference. This simulates the upper part of an ACCD.

**Figure 3 f3-jetem-9-2-i7:**
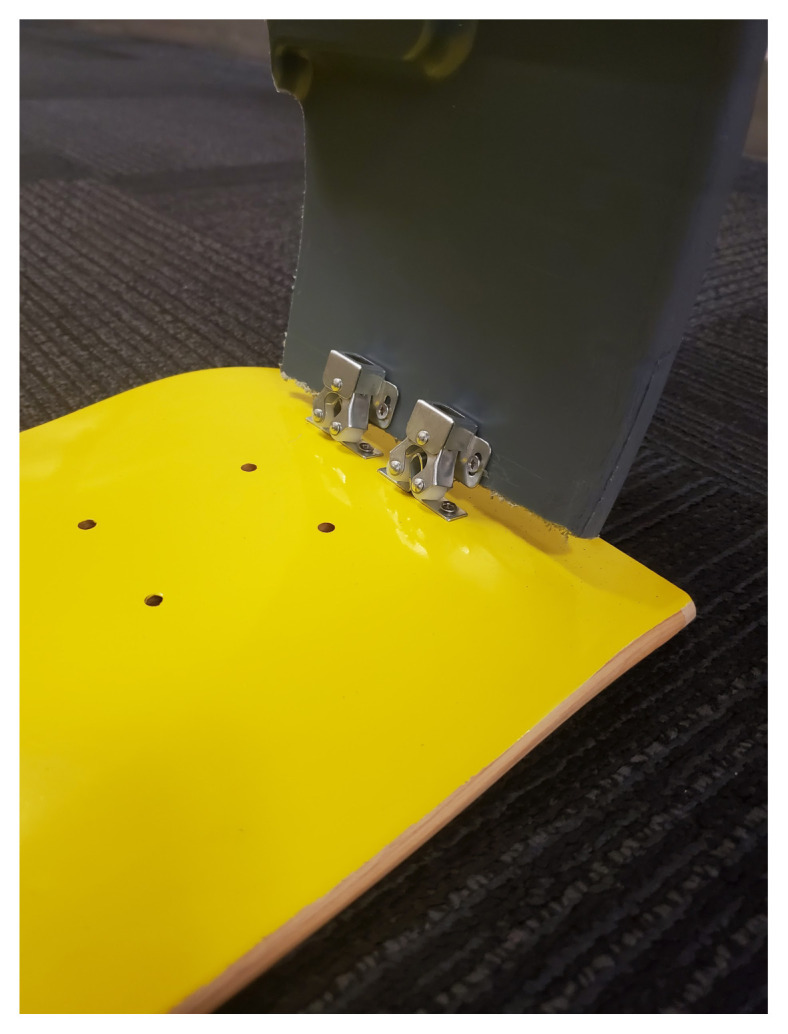
Two latch clips are attached to each side of the arch with screws. The arch is now able to be fastened onto the back plate.

**Figure 4 f4-jetem-9-2-i7:**
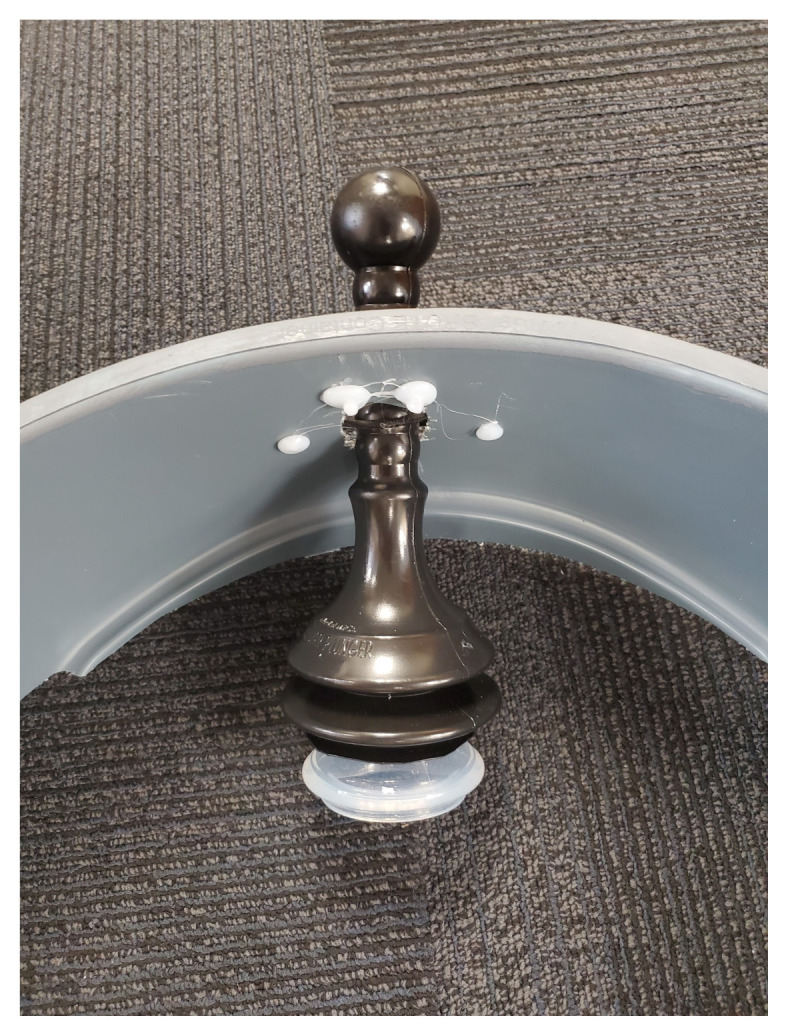
A silicone vacuum suction cup is placed inside the bottom portion of a plunger.

**Figure 5 f5-jetem-9-2-i7:**
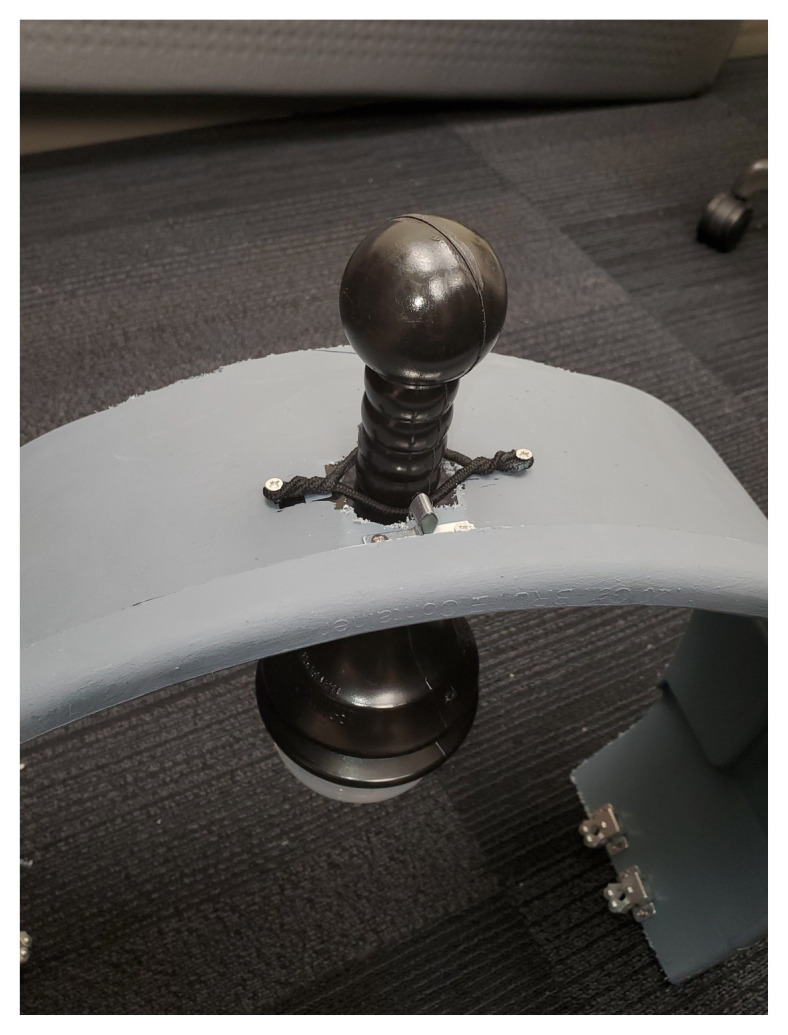
The plunger is suspended through a 2 inch hole on the top portion of the arch. It is held in place by a thick rubber elastic band that is wrapped around 2 screws that are on each side of the hole. There is also a latch clip glued 1 inch in front of the hole.

**Figure 6 f6-jetem-9-2-i7:**
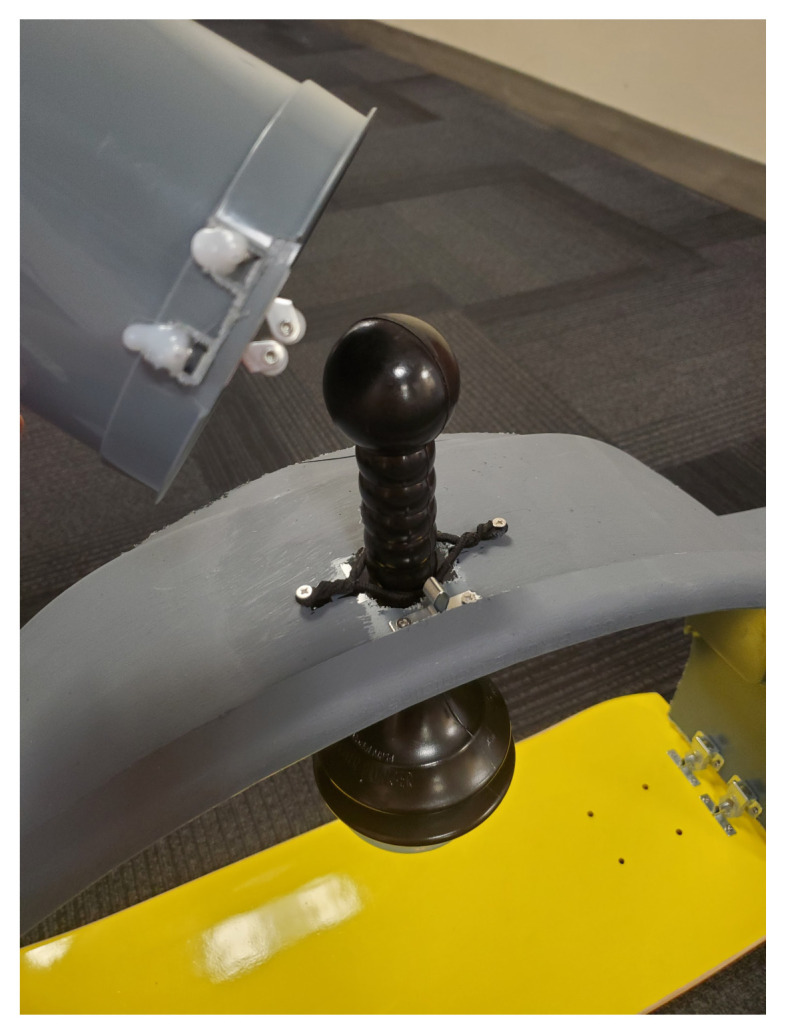
A latch clip is glued on inside portion of a pitcher. It can then be attached to the top portion of the arch and cover the handle of the plunger.

**Figure 7 f7-jetem-9-2-i7:**
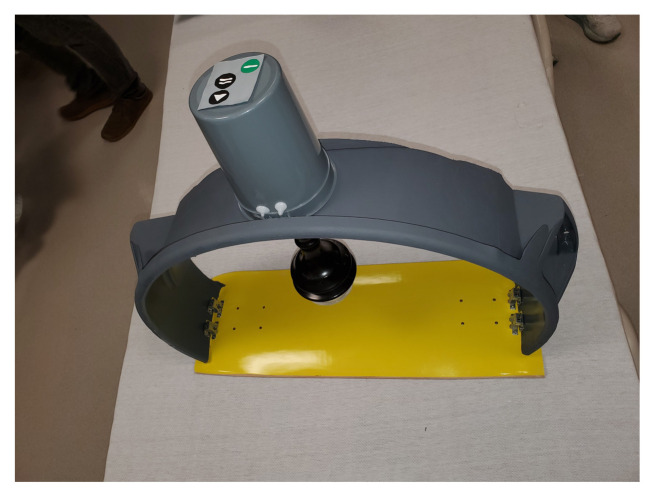
Painted “buttons” are placed on top of the pitcher. Learners press these buttons to simulate turning the ACCD on and off during a cardiac arrest simulation.

**Figure 8 f8-jetem-9-2-i7:**
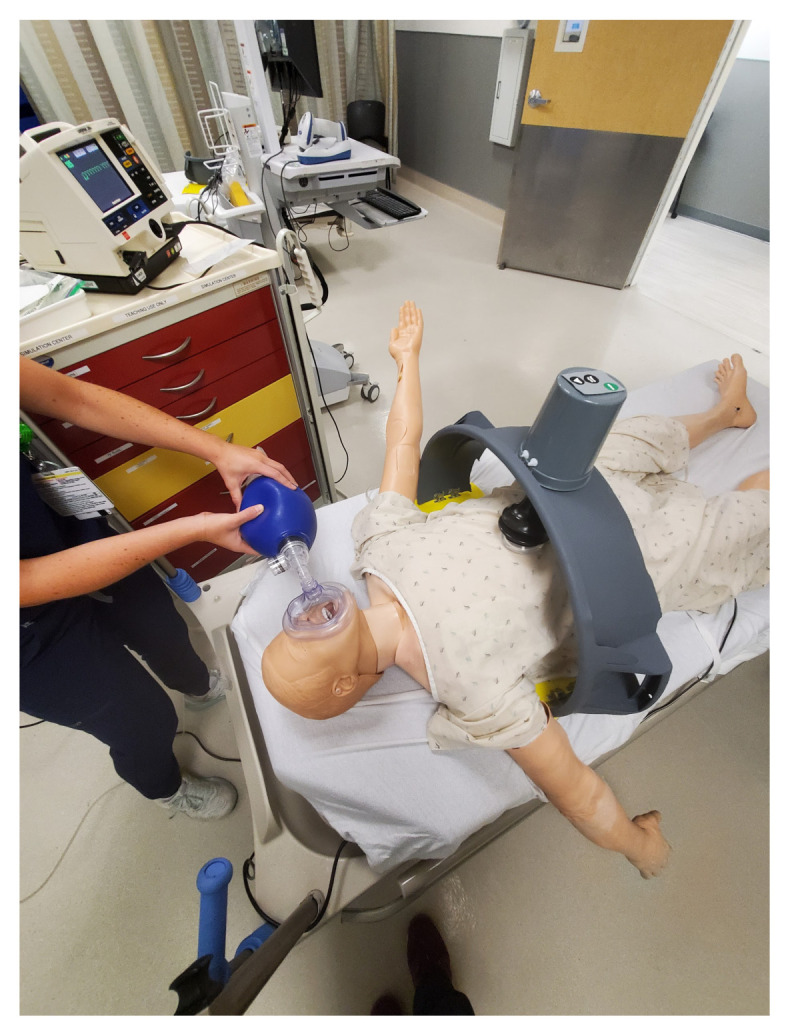
During the simulation, learners clip the upper arch onto the back plate. A bluetooth speaker that simulates the sound of an ACCD is discreetly hidden near the manikin. Learners press the buttons to prompt facilitators to play or turn off the sound.

## LEARNER MATERIALS

[Fig f9-jetem-9-2-i7] [Fig f10-jetem-9-2-i7]
